# The impact of climate change on crop mix shift in the Nordic region

**DOI:** 10.1038/s41598-023-29249-w

**Published:** 2023-02-20

**Authors:** Doan Nainggolan, Abrha Teklay Abay, Jesper Heile Christensen, Mette Termansen

**Affiliations:** 1 Department of Environmental Science, Aarhus University, Frederiksborgvej 399, 4000 Roskilde, Denmark; 2 iCLIMATE Aarhus University Interdisciplinary Centre for Climate Change, Roskilde, Denmark; 3 Department of Food and Resource Economics, University of Copenhagen, Rolighedsvej 23, 1958 Frederiksberg C, Denmark

**Keywords:** Climate-change adaptation, Climate-change impacts, Environmental economics

## Abstract

Growing evidence of anthropogenic climate change suggests marked changes in agricultural ecosystems and crop suitability across the globe. Northern Europe is primarily predicted to see beneficial impacts through crop shifts towards the North of the region. However, studies that quantify the magnitude of climate induced past shifts and the likely future shifts in the agricultural land use patterns are lacking. We use a rich municipality level longitudinal data set from the Nordic region from 1979 to 2012 to study farmers’ adaptation to climate change in terms of crop mix shift. We model four land use classes, namely, cereal, grass, oil seed, and ‘others’, a category summing the remaining agricultural land uses. On top of climatic variables, we include biophysical and economic variables as controls in the regression. We utilize a multinomial fractional logit regression to estimate changes in the land use mix. The projection results indicate that both the near future (2041–2070) and the far future (2071–2100) projected climate are likely to increase the area share of cereal and at the same time decrease the share of grass in the Nordic region relative to the baseline climate (1981–2010). However, these results vary across the region. The results generally suggest a moderate climate induced impact on the spatial crop distributions. Our projection results show a moderate shift in agricultural crop distributions depending on the climate scenario and the time-horizon. Depending on the climate change scenario, grass and cereal are expected to shift by up to 92.8 and 178.7 km, respectively, towards opposite directions; grass towards the South–West and cereal towards the North–East. Overall, the projected areal expansion of cereal towards the North–East is expected to lead to increased environmental pressure.

## Introduction

Research into the consequences of climate change on agriculture has attracted significant interest in the scientific community. Previous studies have revealed that climate change is already changing the distribution of agricultural land use^1,2^. In Europe, changes in crop distributions of climate limited crops have indicated that some crops have been introduced to new areas^3^. Moreover, future climate is expected to trigger further changes in the agricultural land use patterns in Europe. Existing literature suggests that Northern Europe will primarily see beneficial impacts through crop shifts towards the North of the region^4^. However, there is a lack of studies that quantify the extent of the expected climate induced future shift in agricultural land use configuration in the Nordic region, which is the focus of the current paper.

Different approaches have been employed to analyse the likely impacts of climate change on various agricultural outcomes. These cover plant science and agronomic approaches and statistical economic approaches; the latter include the Ricardian and land use share approaches. The plant science and agronomic approaches focus on process-based models of plant growth and yield response to climatic variables using simulations in a laboratory setting (e.g., Hatfield^5^) or field experiment data (eg. Olesen et al.^6^ and Franke et al.^7^). The Ricardian approach^8^ uses farm account data and has been applied mostly to estimate the impact of climate change on agricultural revenue or land rent (e.g. Seo et al.^9^ and Van Passel et al.^10^). The land use share approach, also relying on farm accounting data, has been used to estimate changes in agricultural land use distribution in response to a changing climate (e.g. Reilly et al.^1^ and Cho and McCarl^2^). The different models offer complementary insights into climate change impacts and adaptation options in agriculture. The plant science and agronomic approaches offer insights into the impact of climatic variables on plant physiological processes and the consequential farm management challenges. Meanwhile, the statistical economic approaches generate empirical evidence by assessing how farming has responded to climate change, accounting for factors not included in experimental field research, such as variability in economic, political and technical factors across space and time. The Ricardian approach offers insights into the economic impacts of climate change on agricultural production. The approach implicitly assumes a full range of adaptation to climate change, although it does not explicitly model how. In contrast, the land use share approach models how farmers are likely to respond to climate change through changes in agricultural land use patterns. Thus, in this paper, we apply the land use share approach to demonstrate the mechanism by which farmers are likely to adapt to climate change through agricultural land use change using a detailed municipality level data.

Despite the growing academic interest in modelling the consequences of climate change on agricultural systems, most of the literature focuses on the yield of single crops or on agricultural revenue, and there are only a few studies that investigate the impact of climate change on shifts in land use allocation. Prominent examples of such studies are Reilly et al.^1^, Cho and McCarl^2^ and Sloat et al.^11^. The present study contributes further to the literature on climate change impacts on agricultural land use patterns. Unlike previous works, commonly modelling climate using temperature and rainfall, we consider a richer set of climatic variables, which include growing degree days (GDD). Although GDD has not been used in econometric agricultural land use share analysis, this climatic variable has been found to be a good predictor of agricultural crop yield (e.g., Zhang et al.^12^). Our choice of modelling framework is inspired by previous works^1,2,13–16^. In particular, we follow Cho and McCarl’s^2^ exposition and employ the logit link function to estimate a fractional multinomial logit model. This framework is able to capture the trade-off among the various land use shares in response to changes in climatic and non-climatic variables, and therefore offers a tractable methodology for modelling crop shifts by farmers in response to climate change. Since we are only interested in predicting mean land use proportions and not the full distributions of the land use proportions, we can utilize a multinomial generalization of the Papke and Wooldridge^17^ semi-parametric approach which requires only the specification of the conditional mean of the response variable^2,15,18^.

The present paper covers the Nordic region (Denmark, Finland, Norway, and Sweden). Our analysis first asks the question: What climatic and non-climatic factors drive crop distribution patterns across the region? Secondly, we derive projections of future agricultural land use shares based on two future climate scenarios (RCP4.5 and RCP8.5) over two time horizons (2041–2070 and 2071–2100), and then calculate the changes in agricultural land use distributions and the resulting geographical shifts in land use relative to a baseline climate (1981–2010). The present study addresses the scientific gaps arising from the lack of studies that provide estimates on the extent of climate change impacts on agriculture and the spatial variability across the Nordic region.

## Data and estimation procedures

This study utilizes a comprehensive municipality level data to anaylse the effect of climate change on agricultural land use share. The agricultural land use categories consist of the planted area share of cereals, grass, oil seed, and others. The cereals category includes wheat, barley, oats, rye, triticale, and mixed grain. The grass category constitutes grass and green fodder in rotation, permanent grassland, set aside with grass, grassland under 5 years, reed canary grass, grassland at least 5 years, crops for green fodder and silage, meadows for mowing and pastures, hayfields and silage, hay and pasture. Oil seed category includes rape, linseed, and flax. The others category includes any crop not belonging to the other three categories, nursery area, kitchen garden, unspecified arable land and fallow area. The agricultural land use share data is extracted from the Statistics agencies of each country over the years of 1979–2012. The data covers an unbalanced panel of 1069 municipalities, as individual countries have not collected data over the same time interval, yielding a total of 14,753 observations.

The explanatory variables in this study include both climatic and non-climatic factors, which consist of soil types, agricultural field slope (topography), and crop prices. The climate data is provided by Centro Euro-Mediterraneo sui Cambiamenti Climatici (CMCC) and it contains annual growing degree-days, annual growing season length, annual total precipitation, annual total evapotranspiration, and growing season average soil moisture. The slope variable is derived from Shuttle Radar Topography Mission (SRTM) elevation data, which was accessed through the U.S. Geological Survey website. The soil data, at 500 m $\times$ 500 m grid scale, comes from the EU Joint Research Centre-European Soil Data Centre (ESDAC). The soil data set includes information about soil texture and content of coarse fragment. Soil texture is comprised of percentage of clay, sand, and silt soil. The crop prices data is extracted from FAO statistics and it contains the price of cereals, oil seed, and potato. We merge the land use share data with the historical climate, soil types, agricultural field slope (topography), and crop prices data. The definitions and summary statistics of the variables used in the regression can be found in Table S2 and Table S3, respectively, in the supplementary material online.

We utilize the fractional multinomial logit model to estimate municipality level agricultural land use share as a function of climatic variables and other controls using the unbalanced panel data. We assume that farmers’ land use allocation in the current year is affected by the climate patterns in the immediate previous years and, therefore, our climatic variables are included as lagged 8-year moving average in the model. Moreover, we model the climatic variables with linear and quadratic terms to account for the non-linear relationship between agricultural land use and climate as suggested in the literature (e.g., Cho and McCarl 2017)^2^. Furthermore, our model specification controls for non-climatic variables, which consist of land slope, soil type, and commodity prices. Crop prices are also included as 1-year lags in the regression as we assume that current land use allocation is affected by crop prices in the immediate previous year. On top of climatic and non-climatic variables, the model specification also includes country specific time trends and country fixed effects. The country specific time trend serves as a proxy for other factors that might have influenced change in the patterns of agricultural land use in each country over time (e.g. technological developments), while the country fixed effects factor in differences between countries due to conditions that are specific to each country (e.g. agricultural and environmental policy differences). The standard errors in our regression are clustered by municipality to allow for serial correlation of land use share within municipality. Once we estimate our model, we calculate the marginal effect at mean estimates, holding all the explanatory variables at their long-term (1979–2012) average, as the estimated fractional multinomial parameters do not allow straightforward interpretation.

Furthermore,  given the estimated parametres, we use the estimated relationship between the climatic variables and agricultural land use shares to project the effect of climate change on agricultural land use mix as well as the geographical shift of the land use mix under different future climate change scenarios relative to the baseline climate (1981–2010). The baseline data is, therefore, set as a 30-year average (1981–2010) of all the explanatory variables in the fractional multinomial logit model. The projected climate data, also provided by CMCC, is based on the outputs of the latest version of RCA4 model adopted in the Coupled Model Intercomparison Project Phase 5 (CMIP5) developed at the Rossby Centre, the climate modelling research unit of the Swedish Meteorological and Hydrological Institute (SMHI). The projected climate data is also set to 30-year average for two future projection periods: 2041–2070 and 2071–2100. We conduct our projection analysis based on 1060 municipalities for which data is available both for the baseline scenario and projected climate. Table S4 in the supplementary material online presents the summary statistics of the climate data used in the projection. Specifically, the climate data is used to calculate two related land use projections. First, we calculate projected total changes in agricultural land use share at the Nordic and each country level. The change in agricultural land use share due to climate change is measured by a discrete difference in predicted land use share after and before the change in climate. The baseline and projected climatic variables are averaged at the country and Nordic levels when we estimate the projected land use shares at the country and Nordic levels, respectively. Second, we calculate the weighted average centroid (latitude and longitude) of each agricultural land use for the baseline and future climate scenarios, using long-term average agricultural area in the municipality as a weight. Given the weighted centroids, the effect of climate change on crop mix shift is measured by a discrete difference in centroids after and before the change in climate. In projecting the effects of future climate on agricultural land use patterns, crop prices, soil types, agricultural land slope, and the country specific year variables are kept fixed at their baseline long-term average during the entire projection period.

## Results

### Drivers of current shares of agricultural land uses in the Nordic countries

The parameter estimates from the multinomial regression model do not allow straightforward interpretation. Therefore, in Table 1, we report the marginal effect at mean estimates of the different predictors on the share of each of the four agricultural land use categories. The multinomial fractional logit parameter estimates are reported in Table S5 in the supplementary material online. The regression equations include the following climatic variables: GDD, precipitation, evapotranspiration, and soil moisture. We also run a regression with growing season length (GSL) as an additional predictor, but we had to drop it due to its high correlation with growing degree days (GDD). Previous studies have confirmed that using GDD as a predictor offers an effective alternative to address the non-linear effects of temperature when modelling the impacts of climate change on agricultural land use patterns (e.g. Fezzi et al.^19^, Zhang et al.^12^). We use a threshold temperature of 5 $^{\circ }$C for the calculation of GDD in line with other studies (e.g. Peltonen-Sainio and Jauhiainin^20^ from Finland). We tested the prediction capacity of our model using a hold-out sample within our data (see Figure S3 in the supplementary material online).

Based on the marginal effect at mean estimates reported in Table 1, GDD appears to be the most influential climatic factor in shaping agricultural land use patterns in the Nordic region. GDD is found to be statistically significant predictor for all land use shares except the others category. While increasing GDD exerts favorable influence on the shares of crops (cereal, oil seed), it does on the contrary induce shrinkage in the share of grassland. This suggests that in general a warmer climate tends to drive up crop production, particularly cereal, at the expense of the share of grassland. For example, at the means of all covariates, an increase in GDD by 1 degree day during the growing season is likely to decrease the share of grass by 0.080 percentage points and increase the share of cereal by 0.064 percentage points. The average growing season length in our data is 151.1 days and this means, on the margin, a uniform daily increase of GDD by 1 degree day during the growing season is likely to decrease the share of grass by 12.1 percentage points and increase the share of cereal by 9.7 percentage points.

**Table 1:** Marginal effect at mean estimates.

Variables	Cereal	Grass	Oil seed	Others
GDD	0.00064$$^{***}$$	$$-$$ 0.00080$$^{***}$$	0.00000013$$^{***}$$	0.000058$$^{*}$$
	(8.10)	($-$ 9.41)	(8.35)	(1.76)
Precipitation	$$-$$ 0.00016$$^{***}$$	0.00020$$^{***}$$	$$-$$ 0.000000023	$$-$$ 0.000045$$^{**}$$
	($-$ 2.65)	(3.36)	($-$ 1.33)	($-$ 2.36)
Evapotranspiration	$$-$$ 0.0010$$^{***}$$	0.0011$$^{***}$$	$$-$$ 0.000000067$$^{*}$$	0.0000086
	($-$ 5.65)	(5.73)	($-$ 1.91)	(0.13)
Soil moisture	0.00052$$^{***}$$	$$-$$ 0.00046$$^{***}$$	$$-$$ 0.000000038$$^{*}$$	$$-$$ 0.000038
	(4.44)	($-$ 3.90)	($-$ 1.85)	($-$ 0.86)
Slope	$$-$$ 0.033$$^{***}$$	0.033$$^{***}$$	$$-$$ 0.0000030$$^{***}$$	0.0037$$^{*}$$
	($-$ 6.79)	(6.51)	($-$ 2.75)	(1.85)
Coarse	$$-$$ 0.0086$$^{**}$$	0.010$$^{***}$$	$$-$$ 0.0000022$$^{***}$$	0.00075
	($-$ 2.55)	(2.61)	($-$ 3.30)	(0.66)
Clay	0.0094$$^{***}$$	$$-$$ 0.010$$^{***}$$	0.0000011$$^{**}$$	$$-$$ 0.00041
	(3.53)	($-$ 3.41)	(2.15)	($-$ 0.48)
Sand	$$-$$ 0.0055$$^{***}$$	0.0073$$^{***}$$	$$-$$ 0.0000012$$^{***}$$	$$-$$ 0.0012$$^{**}$$
	($-$ 3.59)	(4.30)	($-$ 4.44)	($-$ 2.36)
Cereal price	0.00062$$^{***}$$	$$-$$ 0.00068$$^{***}$$	$$-$$ 0.000000081$$^{***}$$	0.00022$$^{***}$$
	(11.66)	($-$ 10.49)	($-$ 3.92)	(5.52)
Potato price	0.000072$$^{***}$$	0.000027	$$-$$ 0.000000026$$^{***}$$	$$-$$ 0.00012$$^{***}$$
	(4.14)	(1.16)	($-$ 5.55)	($-$ 7.56)
Oil seed price	$$-$$ 0.00047$$^{***}$$	0.00045$$^{***}$$	0.000000052$$^{***}$$	$$-$$ 0.000056$$^{***}$$
	($-$ 19.11)	(15.35)	(5.08)	($-$ 2.97)
Country dummy	Yes	Yes	Yes	Yes
Country time trend	Yes	Yes	Yes	Yes
Observations	14,753	14,753	14,753	14,753

This table presents the calculated marginal effects based on the estimation results of the pooled multinomial fractional logit model. Robust clustered z-statistics (calculated via the delta method ) are in parentheses. ***, ** and * denote significance on the 1%, 5% and 10% significance level, respectively.

Precipitation, evapotranspiration, and soil moisture exhibit statistically significant relation with the shares of cereal and grass, albeit in contrasting directions of influence. Precipitation and evapotranspiration display positive effect on grass and negative effect on cereal. On the contrary, soil moisture has positive influence on cereal production and the opposite effect on grassland. These results indicate that while water availability in soil is important for cereal production, wetter climate is suitable for grassland but not for cereal production. Moreover, precipitation is also found to be statistically significant climatic determinant for the areal share of others category. The results suggest that increased precipitation tends to reduce the share of this agricultural land use category. In Figure S4 in the supplementary material online, we present a figure of the predicted shares of the various land use classes over the observed range of the climatic variables.

The estimation results show that the area shares of most agricultural land use categories are statistically significantly related with the topography of agricultural areas. The results indicate that crop production (cereal and oil seed) takes place in relatively flat areas while grasslands predominantly occupy agricultural areas with increasing slope. The results also show that soil characteristics are statistically significant factors for the allocation of agricultural land for different agricultural land use categories. The results indicate that agricultural lands with sandy and coarse texture soils are suitable for grass but not for crop production (cereal and oil seed). On the contrary, agricultural lands with clay soils are suitable for cereal and oil seed farming but not for grasslands. This suggests that livestock production relying on grasslands is unlikely to thrive in clay dominated agricultural areas. Moreover, the estimates indicate that the share of others category of agricultural land use appears to be less likely found in areas with sandy soil.

We found that crop market prices are also statistically significantly related with the shares of the different agricultural land uses. The marginal effect estimate for cereal indicates a positive own price effect, meaning that an increase in cereal price induces allocation of more agricultural lands for cereal production. On the contrary, when the price of cereal increases, the shares of grass and oil seed cultivation tend to go down. The estimates indicate that an increase in potato price tends to exert competing effects on cereal and oil seed agricultural land use but it does not have a statistically significant effect on grass land use. For oil seed, the estimation results show positive own price effect. In addition, the results indicate that increase in oil seed price exhibits negative effect on the land use share of cereal but triggers an increase in the land use share of grassland.

### Projected agricultural land use shifts under future climate scenarios

**Figure 1:**
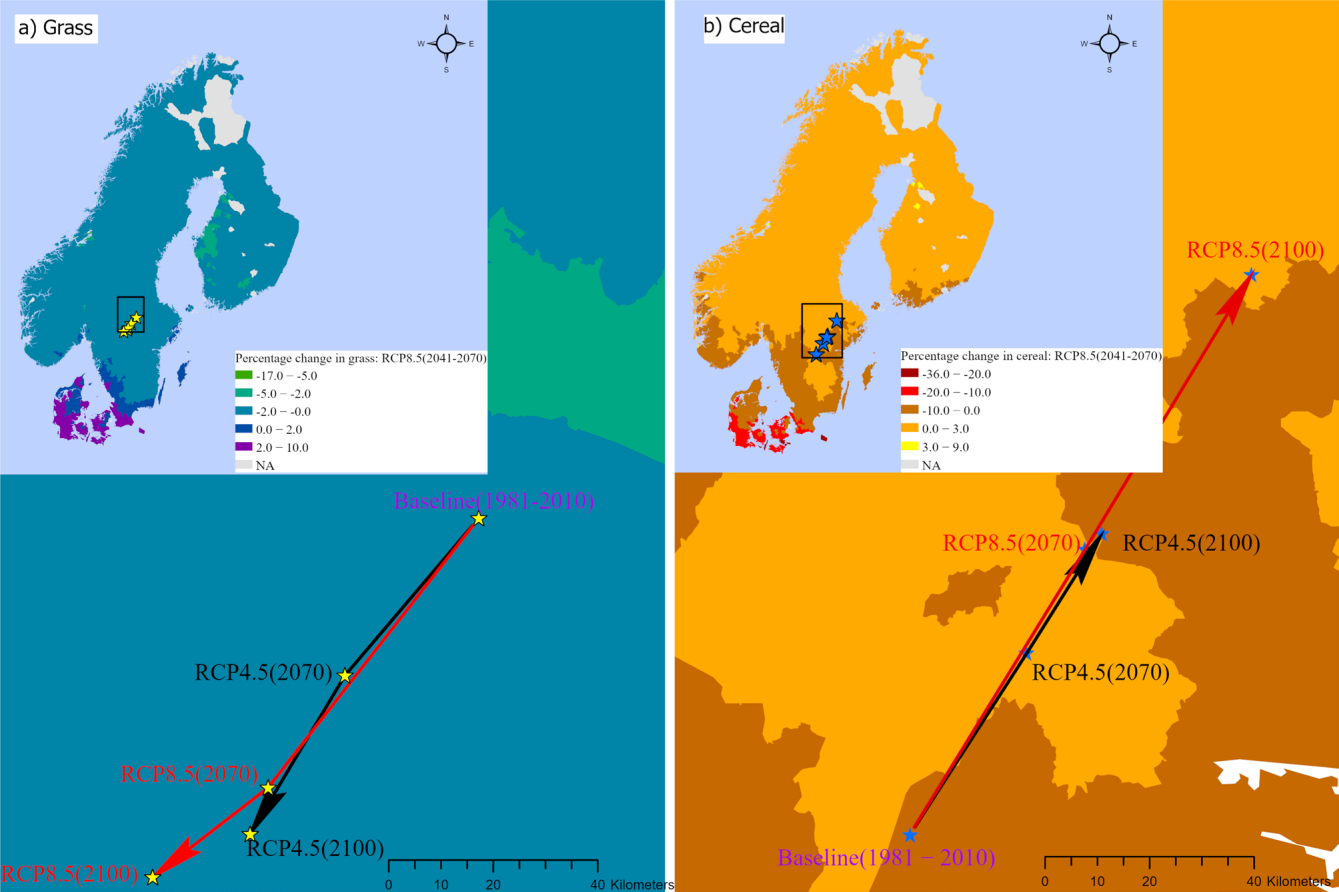
Predicted geographical land use shift for grass and cereal. The main map (top left corner in both (**a**) and (**b**)) presents a weighted municipality level change in land use share under the RCP8.5 (2041–2070) climate relative to the baseline climate scenario.The stars, yellow for grass and blue for cereal, on the directional lines show the weighted centroid of each land use under the different climate scenarios. The red and the black lines show the direction and magnitude of the geographical shift of each land use under the RCP8.5 and the RCP4.5 scenarios, respectively. These maps were generated in ArcGIS Pro 2.6.2 (https://www.esri.com/en-us/arcgis/products/arcgis-pro/overview).

Future climate may potentially bring about some changes in the area distribution of the different agricultural land uses in the Nordic countries. The extent of future projection of climate-driven changes in the agricultural land use shares is varied according to both (1) future climate scenarios (RCP4.5 versus RCP8.5), and (2) the time-horizons taken into account in the analysis. The future climate driven land use changes in the Nordic region entail an expansion of some land use types at the expense of reduced area shares for the other types. We have computed the projected average land use distribution change as well as the resulting geographical shift in land use distribution across the four land use categories for different climate scenarios, relative to the baseline climate. Table 2 presents the land use distribution changes, and we pay close attention on the changes that are statistically significant. In Table 3, we report the agricultural area weighted changes in the centroids of each of the four land use categories, to demonstrate the extent to which future climate is likely to drive geographical shifts in agricultural land use patterns in the Nordic region. Furthermore, Fig. 1 illustrates the municipality level changes in grass and cereal land use share under the RCP8.5 (2041–2070) scenario, as well as the direction of the geographical shift under all the different scenarios. The predicted changes in shares presented in the main maps of the figures are weighted by the long-term average total agricultural area share in the municipality and, thus, indicate the percentage change for a specific land use category out of the total municipality area due to the change in climate. In addition, we present maps of projected land use shares for cereal and grass under the remaining future climate scenarios in the supplementary material online.

**Table 2:** Predicted change in agricultural land use shares under different climate change scenarios relative to the baseline climate.

Country	Scenario	Cereal	Grass	Oilseed	Others
Nordic	RCP4.5 (2041–2070)	0.0401$$^{***}$$	$$-$$ 0.0515$$^{***}$$	0.0000253$$^{***}$$	0.0113$$^{***}$$
		(4.80)	($-$ 6.21)	(6.65)	(4.06)
	RCP8.5 (2041–2070)	0.0502$$^{***}$$	$$-$$ 0.0675$$^{***}$$	0.0000371$$^{***}$$	0.0173$$^{***}$$
		(4.20)	($-$ 5.75)	(5.74)	(4.22)
	RCP4.5 (2071–2100)	0.0485$$^{***}$$	$$-$$ 0.0685$$^{***}$$	0.0000411$$^{***}$$	0.0199$$^{***}$$
		(3.74)	($-$ 5.36)	(5.55)	(4.50)
	RCP8.5 (2071–2100)	0.0649$$^{**}$$	$$-$$ 0.122$$^{***}$$	0.000110$$^{***}$$	0.0575$$^{***}$$
		(2.23)	($-$ 4.54)	(4.49)	(5.00)
Denmark	RCP4.5 (2041–2070)	$$-$$ 0.108$$^{***}$$	0.0174	0.0307$$^{***}$$	0.0595$$^{***}$$
		($-$ 5.70)	(1.15)	(2.78)	(5.60)
	RCP8.5 (2041–2070)	$$-$$ 0.176$$^{***}$$	0.0478$$^{**}$$	0.0334$$^{*}$$	0.0951$$^{***}$$
		($-$ 6.84)	(2.08)	(1.88)	(5.73)
	RCP4.5 (2071–2100)	$$-$$ 0.171$$^{***}$$	0.0357	0.0387$$^{**}$$	0.0966$$^{***}$$
		($-$ 5.81)	(1.46)	(2.00)	(5.20)
	RCP8.5 (2071–2100)	$$-$$ 0.351$$^{***}$$	0.0225	0.0729	0.255$$^{***}$$
		($-$ 5.22)	(0.40)	(1.24)	(3.62)
Finland	RCP4.5 (2041–2070)	0.0633$$^{***}$$	$$-$$ 0.0770$$^{***}$$	0.0139$$^{***}$$	$$-$$ 0.000197
		(8.13)	($-$ 9.61)	(7.77)	($-$ 0.06)
	RCP8.5 (2041–2070)	0.0864$$^{***}$$	$$-$$ 0.109$$^{***}$$	0.0219$$^{***}$$	0.000712
		(7.43)	($-$ 9.41)	(7.56)	(0.16)
	RCP4.5 (2071–2100)	0.0847$$^{***}$$	$$-$$ 0.113$$^{***}$$	0.0253$$^{***}$$	0.00333
		(6.78)	($-$ 9.02)	(7.74)	(0.68)
	RCP8.5 (2071–2100)	0.0930$$^{***}$$	$$-$$ 0.180$$^{***}$$	0.0607$$^{***}$$	0.0264$$^{**}$$
		(3.41)	($-$ 7.60)	(6.61)	(2.17)
Norway	RCP4.5 (2041–2070)	0.0380$$^{***}$$	$$-$$ 0.0546$$^{***}$$	4.31e$$-$$10	0.0166$$^{***}$$
		(3.61)	($-$ 4.69)	(0.08)	(3.99)
	RCP8.5 (2041–2070)	0.0660$$^{***}$$	$$-$$ 0.0894$$^{***}$$	7.03e$$-$$10	0.0235$$^{***}$$
		(4.47)	($-$ 5.48)	(0.02)	(3.78)
	RCP4.5 (2071–2100)	0.0459$$^{***}$$	$$-$$ 0.0719$$^{***}$$	6.38e$$-$$10	0.0260$$^{***}$$
		(2.96)	($-$ 4.21)	(0.02)	(4.02)
	RCP8.5 (2071–2100)	0.0706$$^{**}$$	$$-$$ 0.137$$^{***}$$	1.72e$$-$$09	0.0662$$^{***}$$
		(2.57)	($-$ 4.47)	(0.01)	(4.35)
Sweden	RCP4.5 (2041–2070)	0.0204$$^{**}$$	$$-$$ 0.0432$$^{***}$$	0.00970$$^{***}$$	0.0131$$^{***}$$
		(2.09)	($-$ 4.15)	(8.40)	(3.55)
	RCP8.5 (2041–2070)	0.0145	$$-$$ 0.0474$$^{***}$$	0.0134$$^{***}$$	0.0195$$^{***}$$
		(0.98)	($-$ 3.05)	(6.79)	(3.44)
	RCP4.5 (2071–2100)	0.0155	$$-$$ 0.0567$$^{***}$$	0.0158$$^{***}$$	0.0255$$^{***}$$
		(0.99)	($-$ 3.43)	(7.40)	(4.17)
	RCP8.5 (2071–2100)	$$-$$ 0.0150	$$-$$ 0.108$$^{***}$$	0.0387$$^{***}$$	0.0838$$^{***}$$
		($-$ 0.40)	($-$ 2.95)	(5.00)	(4.54)

This table presents the change in agricultural land use shares for different climate scenarios and time horizons. Robust z-statistics are reported in parentheses. The standard errors used to calculate the z-statistics are estimated using the delta method. ***, ** and * denote significance on the 1%, 5% and 10% significance level, respectively.

In general, under future climate scenarios, the Nordic region is expected to see increased shares of cereal and decreased share of grassland. Our findings indicate that the magnitude of the changes under the RCP8.5 is more prominent than it is under the RCP4.5. Table 2 shows that, depending on the future climate scenarios and time horizons, the share of cereal for the whole Nordic region is expected to increase  by between 4 and 6.5 percentage points. For grass, the reduction in its share is estimated to be between 5.1 and 12.2 percentage points. The share of the others category is expected to go up by between 1.1 and 5.8 percentage points. Our projection analysis is intended to illustrate the impact of climate change on land use distribution using plausible projections of future climate from a single climate model. The results do not account for uncertainties in future climate projections as might have been obtained by using information from different climate models. However, a limited sensitivity study to test aspects of this uncertainty (see Table S6 in the supplementary material) suggests that though there are minor differences in the magnitude of projected changes, our central messages on the patterns of land use shift remain unchanged.

**Table 3:** Predicted geographical crop mix shift (km).

	Cereal$^{\hbox {NE}}$	Grass$^{\hbox {SW}}$	Oilseed$^{\hbox {NE}}$	Others$^{\hbox {SW}}$
RCP4.5 (2041–2070)	58.8	39.5	21.7	59.6
RCP8.5 (2041–2070)	97.3	74.6	51.6	84.1
RCP4.5 (2071–2100)	91.2	65.4	52.8	80.6
RCP8.5 (2071–2100)	178.7	92.8	104.5	128.3

This table presents the geographical shift in the centroids of the four land use classes for different climate scenarios and time horizons relative to the predicted shares under the baseline climate scenario (1981–2010).The superscripts on the land use types indicate the direction of the geographical shift (*NE* North–East, *SW* South–West).

The results in Table 2 also suggest that the impacts of future climate are likely to manifest some differences in agricultural land use patterns between countries in the Nordic region. We highlight changes that are consistently statistically significant across all climate scenarios and time-horizons. For Denmark, future climate driven agricultural land use changes are expected to be dominated by reduction in the areal share of cereal land use. Depending on the climate scenario and time-horizon, land allocation for cereal is expected to reduce by between 10.8 and 35 percentage points. The decrease in areal share of cereal primarily manifests as a compensation for a marked increase in areal share of the others category by between 6 and 25.5 percentage points. For Finland, future climate is likely to trigger land use competition between grassland (reduction in share) and both cereal and oil seed (expansion in share). The reduction in grass is expected to range between 7.7 and 18 percentage points. The expansion is expected to range between 6.3 and 9.3 percentage points in the case of cereal and between 1.4 and 6.1 percentage points in the case of oil seed. Norway is also expected to see an expansion of cereal cultivation (between 3.8 and 7.1 percentage points) and a decrease in grass (between 5.5 and 13.7 percentage points). In addition, the areal share of the others category is also expected to increase in Norway, ranging from 1.7 to 6.6 percentage points. For Sweden, changes in agricultural land use due to a change in future climate are likely to be marked by some degree of land substitution between grassland (decrease in share) and oil seed and others (increase in shares). Depending on the climate scenarios and time-horizons, areal share of grass is expected to go down by between 4.3 and 10.8 percentage points. Oil seed and others are expected to increase by between 1 and 3.9 percentage points and between 1.3 and 8.4 percentage points, respectively. All in all, in relative terms, considering the total agricultural area in each of the countries in Nordic region, the results indicate that the most pronounced changes in agricultural land use patterns under future climate scenarios are likely to occur in Denmark.

Regarding geographical shift, our findings indicate a moderate shift in the spatial distribution of agricultural land uses across the Nordic region. The extent of the geographical shift varies depending on the climate scenario and the time-horizon. In general, the estimates in Table 3 indicate that more pronounced shifts are likely to occur under the RCP8.5 climate scenario and for the far future time horizon (2071–2100). To illustrate, for the far future time horizon, the centroids of cereal and oil seed are projected to move towards the North–East by 178.7 km and 104.5 km, respectively, under the RCP8.5 climate scenario, while the change is only 91.2 km and 52.8 km, respectively, under the RCP4.5 climate scenario. On the contrary, climate change is projected to drive the geographical centriod of grass land by 92.8 km towards the South–West under the RCP8.5 climate change scenario and for the far future time horizon, while this change under the RCP4.5 climate scenario is projected to be only 65.4 km. The movement of grass towards the South-West is also confirmed in Fig. 1, which is based on the RCP8.5 (2041–2070) climate scenario, which shows an increase in the area share of grass in the south of the region and a decrease in the north. Interestingly, considering the far future time horizon, the difference in geographical shift between the RCP4.5 and RCP8.5 for grass is not as pronounced as for cereal.

## Discussion and conclusion

### Climate change, geographical shifts and agricultural land use trade-offs

Most of the previous studies, and studies from northern Europe in particular, have focused on modelling climate change impact on the agricultural land value or changes in crop yield^8,12,21–23^. Furthermore, many of the existing studies only deal with individual crops hence lacking insights into land use interrelationships—how changes in land area of one crop may affect allocation of land for other crop production. Using a land use share modelling framework, this research provides insights into the extent of land use trade-offs between different agricultural crops due to future changes in the climate regime. The present study therefore has potential to contribute to better understanding of land allocation which is an important form of adaptation to climate change^24,25^. For the Nordic region as a whole, as the present study shows, under future climate, area share of grass is expected to decrease by as much as 12.2 percentage point while cereal and others are expected to expand by as much as 6.5 and 5.8 percentage points respectively. However, the change in the agricultural land use varies between the North and South of the region, resulting in shifts in the spatial distribution of agricultural land use under a future climate. As an example, depending on the climate scenarios, grass is expected to shift between 39.5 and 92.8 km towards the South–West while cereal is predicted to shift between 58.8 and 178.7 towards North–East. The change in longitude and latitude for all crops is presented in Table S7 in the online supplementary material.

Agricultural land use shifts can have important environmental implications, for example, in terms of CO$_{2}$ emissions. For example, Denmark’s 2019 greenhouse gas inventory submitted to the United Nations Framework Convention on Climate Change (UNFCCC) shows that, the spatial distribution of land use change in agriculture is important for achieving CO$_{2}$ mitigation targets. The conversion of 37,400 ha of grassland on mineral soil to cropland is reported to result in net CO$_{2}$ emissions of 25,170 tonnes. On the contrary, the conversion of 79,980 ha of cropland to grassland on mineral soil is reported to result in a net removal of 38,440 tonnes of CO$_{2}$^26^. However, assessing the effect of CO$_{2}$ emissions on land use change is not within the scope of this paper. It is also likely that the conversion of grassland into cereals and other crops would result in increased use of agricultural inputs, such as fertilizer and pesticides, which could have negative impact on water quality and other forms of environmental pollution.

### Climate change scenarios and time horizons

As highlighted by Zhang et al.^12^, despite the growing number of studies assessing the consequences of climate change on agriculture, large proportion of the studies have been limited to modelling the effects of a narrow set of climatic variables, mainly temperature and precipitation. Our study demonstrates that the configuration of agricultural land use shares in the Nordic region is influenced by a wide range of climatic variables, including growing degree days. In addition, our research highlights the importance of controlling for non-climatic variables in the modelling. In this study, we include soil properties, land topography, crop prices, and country level dummies and linear time trends. Controlling for the influence of these non-climatic factors leads to a more realistic estimation of the likely consequences of future climate regimes at different geographical and temporal scales. The chosen climate projection and the temporal spans within which the effects are being quantified are some of the most important sources of variation in the estimates of the magnitude of the effects of climate change on agriculture^4,23^. In this study, we consider two future climate scenarios (RCP4.5 and RCP8.5) and assessed the effects on agricultural land use shares for two time horizons (2041–2070 and 2071–2100). Not surprisingly, our findings indicate that for the Nordic region, the most striking effects of climate change on agriculture will occur under future climate regime that is consistent with the RCP8.5 climate projection, and particularly in the far future time horizon (2071–2100). The magnitude of change in area share of grass under RCP8.5 in the far future time horizon (2071–2100) is more than double the magnitude of the change under RCP4.5 in the near future (2041–2070). In the case of others category, the magnitude of change under the RCP8.5 scenario for 2071–2100 projection period is about five times the magnitude under RCP4.5 in the near future time horizon (2041–2070). The more pronounced effect of future climate under RCP8.5 in the far future time horizon compared to RCP4.5 in the near future can also be seen with regard to the geographical shifts. To illustrate, under RCP8.5 in the far future time horizon, cereal is expected to geographically shift three times as far as it is under RCP4.5 in the near future time horizon.

### Disparities between countries in the Nordic region

A systematic review of the reported challenges and opportunities in agriculture due to climate change in the Nordic region and potential adaptation actions has concluded that benefits from climate change are expected for this region^27^. The review gives examples of a climate change impact gradient from South–West to North–East in line with the findings in this research. The examples include shifts to new crops and changes from spring-sown to winter-sown crops. Other studies have also highlighted the role of altering land allocation as an adaptation response to anticipated shifts in climatic suitability for crop production in the north^23,28,29^. Nonetheless, the findings from the present study highlight that countries across the Nordic region may have different adaptation patterns, including differences in opportunities to benefit from the changing climate. Our findings show that the agricultural land use trade-offs driven by future climate for Denmark is different from that for Finland, Norway and Sweden. For Denmark, farmer adaptation to future climate is expected to manifest itself through reallocation of arable lands presently devoted to cereal production. Meanwhile, for the other countries, adaptation is expected to occur at the expense of grasslands. Overall, most pronounced intensity of shifts in the land use patterns as a consequence of climate change is expected for Denmark. This may suggest two potential explanations. Firstly, the findings show a large conversion from cereal to the others category, which suggests that there is a lot more scope for adaptation via crop reallocation in Denmark compared with the other countries in the Nordic region (e.g., Odgaard et al.^3^). Yet, our analysis did not consider crops that are currently grown south of the study region that might be suitable in future Danish climate. Secondly, the findings also suggest that agricultural land use configuration in Denmark is more sensitive to climate change as it has a warmer baseline climate compared to the other Nordic countries. This implies that for the high-end warming scenario for Denmark, the average climate projection lies outside of the historical data range, making the predictions for Denmark more uncertain than those for the other countries. Furthermore, there might be an increased risk of biased predictions when we predict at the municipality level instead of a regional or national averages, as the projected climate for some municipalities under some warming scenarios lies outside of the historical observed data range used to estimate our model.

The estimated relationship between climatic variables and land use is only unbiased if there are no omitted variables that might explain the changes in land use and are correlated with climate. Moreover, it is important to note that in the present study, our future climate driven land use projections are based on the assumption that both total agricultural area and relative prices remain at their baseline values. However, total agricultural area is likely to change in the future due to factors such as future agricultural and environmental policies (e.g. afforestation), which could  affect the different land uses differently compared to the baseline policies. For example, historical trends  suggest a slight decline in the total area of agricultural land in the Nordic region in the future (see Figure S2 in the supplementary material online). Similarly, the relative prices of agricultural commodities are also likely to  change in the future. Therefore, a comprehensive understanding of the variation in climate- and climate policy induced land use shifts across the Nordic region should take into account both the drivers of change in total agricultural area and the variation in adaptation strategies for alternative agricultural land uses. This warrants further investigation.

## Methods

We apply a multivariate framework with *J* land use types where the response variable of interest denotes a vector of proportions, ${y}\equiv (y_{1}, y_{2},\dots , y_{J})$ which correspond to a set of *J* exhaustive, mutually exclusive land use categories. Since the four land use classes encompass all the possible agricultural land use types, the sum of the land use proportions in a municipality should add up to one. Let $y_{itj}$ denote the fractional response variable defined on the unit interval for municipality *i* at time *t* for land use *j* and let $\bf {x_{it}}$ denote the set of explanatory variables. The fractional regression is given by the following conditional expectation:

1 $$\begin{aligned} E(y_{itj}|\bf{{x_{it}}})=G(\varvec{{x_{it}}\theta _{j}}) \end{aligned}$$

where $\varvec{\theta}$ denotes a vector of parameters of interest and $G(\cdot )$ is a standard cumulative density (CDF). Typically, researchers use either the probit or logit link function for $G(\cdot )$. If one assumes a logistic link function, $G(\cdot ) = \Lambda (\cdot )$, the conditional mean of the $j^{th}$ share is given by^30^:

2 $$\begin{aligned} E(y_{itj}|\bf{{x_{it}}})=\dfrac{\text {exp}(\bf{{x_{it}}\varvec\theta _{j}})}{\big [1+\sum _{h=2}^{J}\text {exp}(\bf{{x_{it}}\varvec\theta _{h}})\big ]},\quad \forall j=1,\dots ,J \end{aligned}$$

The structural parameters, $\varvec{\theta }$, are estimated using a pooled fractional multinomial logit model. As estimating the fractional multinomial logit model requires some normalization, we set the coefficients of the first equation to zero. The estimation is carried out using quasi maximum likelihood estimator (QMLE)^17,31,32^. As we are estimating a pooled regression, the time dimension is disregarded. Thus, the likelihood contribution of observation *i* is given by:

3 $$\begin{aligned} {\mathscr {L}}_{i}(\varvec{\theta} )=\prod _{j=1}^{J}E(y_{ij}|{{\bf x_{i}}})^{y_{ij}} \end{aligned}$$

The parameter vector, $\hat{\varvec{\theta }}$, is a solution to a maximization of the total log-likelihood function which is a sum of individual likelihood contributions.

4 $$\begin{aligned} {\hat{\varvec{\theta }}}= \mathop {\mathrm {arg\,max}}\limits _{\theta }\sum _{i=1}^{N}\sum _{j=1}^{J}y_{ij}\cdot {\text {log}}(E(y_{ij}|\bf{{x_{i}}})) \end{aligned}$$

The quasi-maximum likelihood estimator of $\varvec{\theta}$ is consistent and asymptotically normal since the logit link function is a member of linear exponential family (LEF)^17,31^. In a multinomial fractional logit framework, the estimated structural parameteres can not entirely determine the magnitude and even the sign of the effect of the explanatory variables and thus one should calculate the marginal effects^32^. The computation of the partial effect requires a weighted sum of all other parameters. The partial (marginal) effect of the *k*th continuous covariate on the *j*th land use share is calculated as:

5 $$\begin{aligned} PE_{ijk}=\dfrac{\partial E(y_{ij}|{\bf{{x_{i}}}})}{\partial x_{ik}}=E(y_{ij}|{\bf{{x_{i}}}})\cdot \bigg [\hat{{\theta }} _{jk}-\dfrac{\sum _{h=2}^{J} \hat{{\theta }} _{hk}{\text {exp}}({\bf{{x_{i}}}}\hat{\varvec{\theta }} _{h})}{[1+\sum _{h=2}^{J}{\text{exp}}({\bf{{x_{i}}}}\hat{\varvec{\theta }} _{h})]}\bigg ] = E(y_{ij}|{\bf{{x_{i}}}})\cdot \bigg [\hat\theta _{jk}-\sum _{h=2}^{J} \hat{{\theta }} _{hk}E(y_{ih}|\bf{{x_{i}}})\bigg] \end{aligned}$$

For factor variables, the partial effect estimates are the discrete first difference from the reference (base) category. The marginal effect at mean estimates can be calculated by plugging the mean values of the explanatory variables into the equation, whereas average marginal effect is obtained by taking the average of the marginal effects across all observations.

The predicted change in shares due to climate change is measured as a discrete difference in predicted shares after and before climate change. The non-climatic variables are fixed at their long-term average value when we calculate the predicted changes. Predicted changes in land use shares for municipality *i* and for land use *j*, $\Delta _{ij}({w})$, due to a change in climate variable from $w_{0}$ to $w_{1}=w_{0}+\Delta$ is given by

6 $$\begin{aligned} \Delta _{ij}({w})=E(y_{ij}|{w_{1}}; {\overline{\bf{x}_{i}}}, {\hat{\bf{\varvec\theta }}})-E(y_{ij}|{w_{0}} ;{\overline{\bf{x}_{i}}}, {\hat{\bf{\varvec\theta }}}), \forall j=1,\dots ,J \end{aligned}$$

The predicted agricultural area weighted centroid of agricultural land use *j* for a given climate scenario is given by

7 $$\begin{aligned} {\hat{L}}_{j} ({w})=\sum _{i=1}^{N}\dfrac{A_{i} E(y_{ij}|{w}; {\overline{\bf{x}_{\bf{i}}}}, {\hat{\bf{\varvec\theta }}})}{\sum _{i=1}^{N}A_{i} E(y_{ij}|{w}; {\overline{\bf{x}_{\bf{i}}}}, {\hat{\bf{\varvec\theta }}})}L_{i} \end{aligned}$$

where $A_{i}$ is the long-term average municipality agricultural area and $L_{i} = \{Latitude_{i}, Longitude_{i} \}$ is the centroid of municipality *i*. Given the predicted centroids, the geographical shift of agricultural land use is measured by the discrete difference in the weighted centroids before and after the change in climate.

## Supplementary Information

Supplementary Information.

## Data Availability

The datasets analysed during the current study are available from the corresponding author on request.
