# Supplementary material for: The impact of climate change on crop mix shift in the Nordic region
Source: Sci Rep. 2023 Feb 20;13:2962. doi: 10.1038/s41598-023-29249-w (PMC9941477; doi:10.1038/s41598-023-29249-w)
Supplement: Supplementary file 1 — Supplementary Information. [file 41598_2023_29249_MOESM1_ESM.pdf]

The impact of climate change on crop mix shift in the Nordic region

Doan Nainggolan[1, 2], Abrha Teklay Abay[*, 1, 3], Jesper Heile Christensen[1], and Mette Termansen[3]

[1]Department of Environmental Science, Aarhus University, Frederiksborgvej 399, 4000 Roskilde, Denmark
[2]iCLIMATE Aarhus University Interdisciplinary Centre for Climate Change, Denmark
[3]Department of Food and Resource Economics, University of Copenhagen, Rolighedsvej 23, 1958 Frederiksberg C, Denmark
[*]Correspondence and request for materials should be addressed to A.T.A.(email: aba@envs.au.dk or ata@ifro.ku.dk)

Supplementary Information

S.1 Data

S.1.1 Land use data

Four agricultural land uses are included, namely cereal, grass, oilseeds and "others". The cereals category includes wheat, barley, oats, rye, mixed corn, triticale, and mixed grain. The grass category constitutes grass and green fodder in rotation, permanent grassland, set aside with grass, grassland under 5 years, reed canary grass, grassland at least 5 years, crops for green fodder and silage, meadows for mowing and pastures, hayfields and silage, hay and pasture. Oil seeds category includes rape, linseed, and flax. The others category includes any crop not belonging to the other three categories, nursery area, kitchen garden, unspecified arable land and fallow area. The agricultural land use data is compiled from the Statistic agencies of Denmark, Finland, Norway, and Sweden and spans more than 30 years from 1979 - 2012. The data covers an unbalanced panel of 1069 municipalities due the fact that individual countries have not collected data in the same interval across time and this gives us a total of 14,753 observations. For each country, land use data is available at municipality level, which determines the spatial resolution of our analysis. As such, share of each of the four agricultural land uses is calculated for each municipality as the area occupied by an agricultural land use divided by the total of agricultural land in a given municipality. Figure S1 displays the annual average distribution of the various agricultural land uses by country. It is also important to present the historical trend of agricultural land use, as we are only dealing with agricultural land use, not total land use. Therefore, in Figure S2, we present the historical trend of agricultural land use in the four Nordic countries. Table S1 presents the annual number of observations by country.

Figure S1. Average land use share over time by country

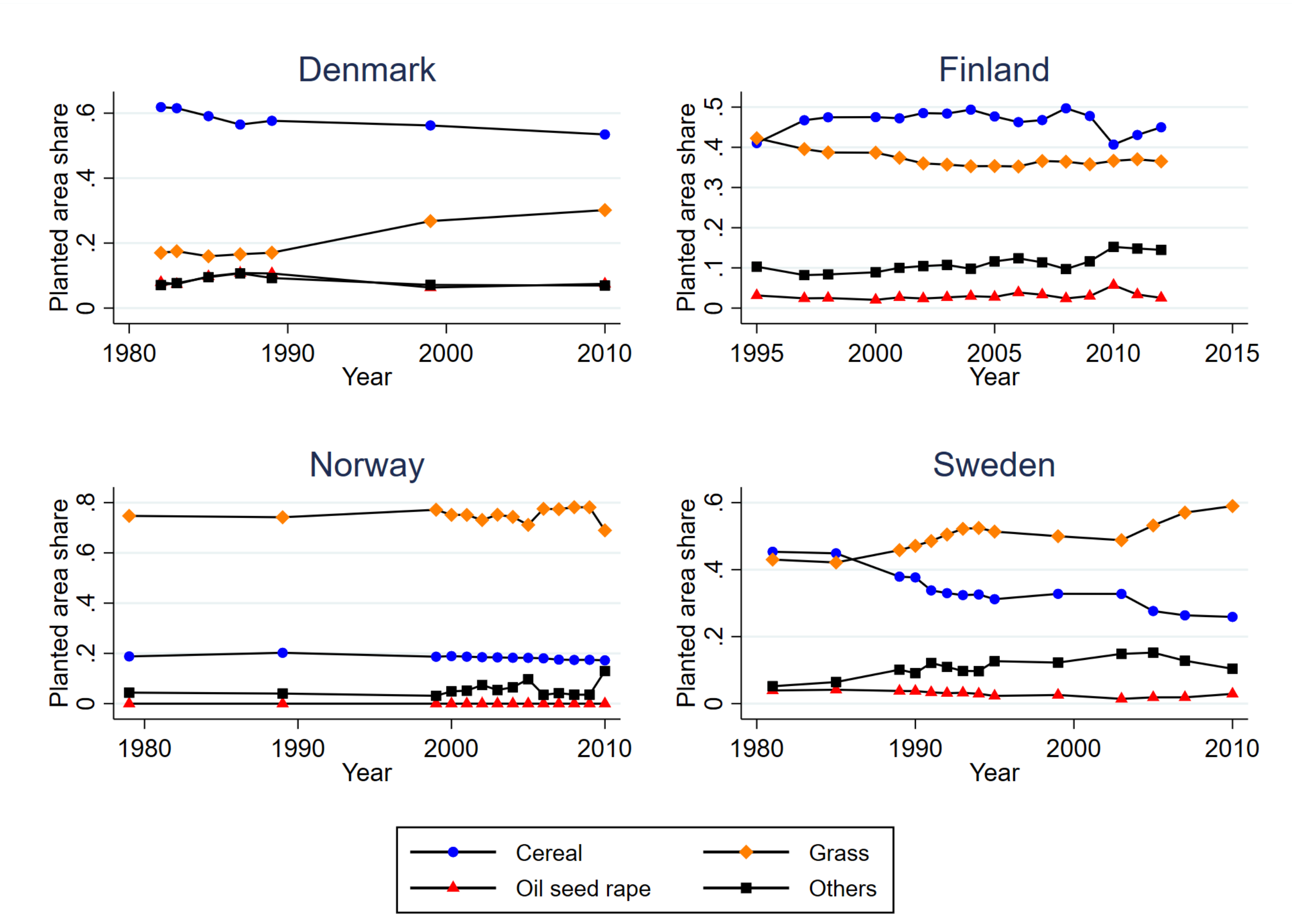

Figure S2. Annual total agricultural area by country

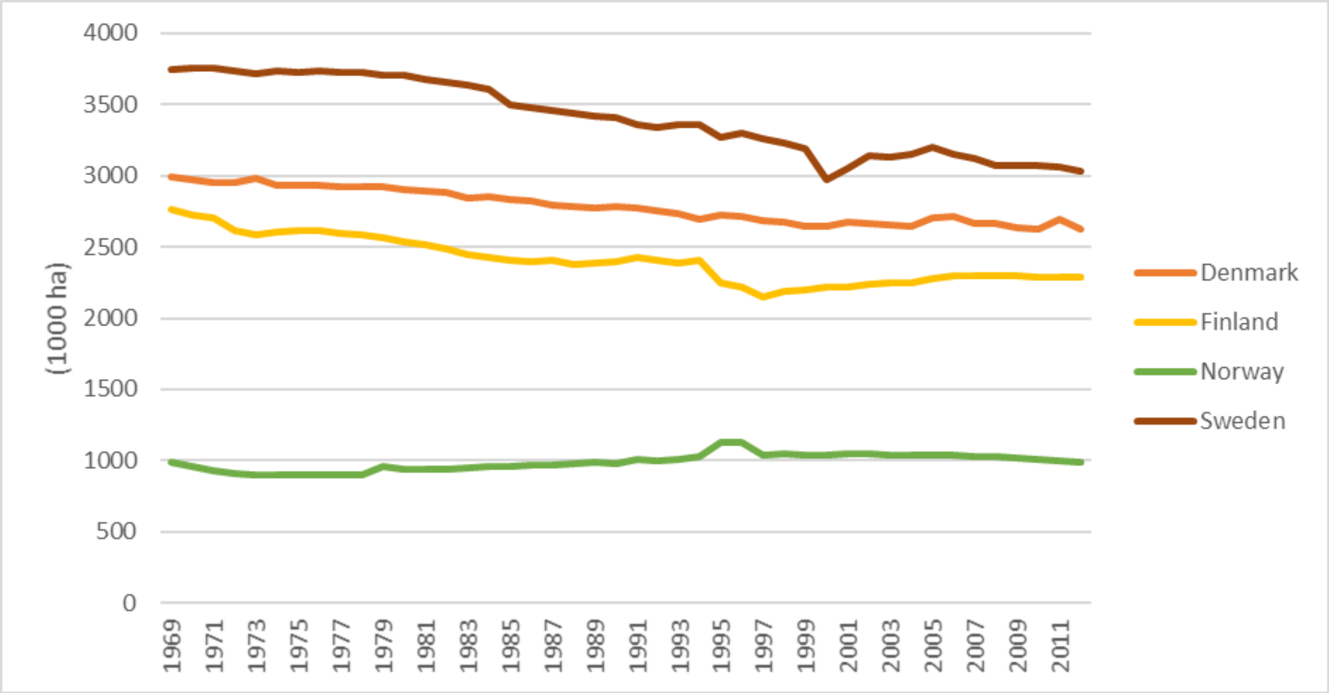

S.1.2 Climate data

Climate data was provided by Centro Euro-Mediterraneo sui Cambiamente Climatici (CMCC). The data is based on the outputs of the latest version of the high resolution Regional Climate Model (RCM) developed at the Rossby Centre, the climate modelling research unit of the Swedish Meteorological and Hydrological Institute (SMHI). The climate data covers several variables and the temporal resolution was daily. For the purpose of the analysis in the present paper, we preprocessed the climate data in order to derive the following variables: annual number of growing degree-days, annual growing season length, total annual precipitation, total annual evapotranspiration, and average soil moisture over growing season (see Table S2 on page 4). While earlier research assessing climate impacts on agricultural land use shifts attempted to capture the non-linear effect of temperature by for example specifying both the linear and quadratic forms in the model estimations[1–3], more recent studies addressed the issue by using temperature data to calculate growing degree days and including this variable in the analysis instead[4,5]. Precipitation, evapotranspiration, and soil moisture variables are included in the present analysis to capture processes determining water availability for agricultural production. The spatial resolution of the original climate data was 14 km x 14 km. In order to match the spatial resolution of the land use data, in ArcGIS, we calculated the average value of a given climate variable for a given municipality.

S.1.3 Non-climate data

Topography and land quality are important factors for the suitability of different agricultural land uses as has been shown in previous agricultural land use share models[6–8]. For the present study, we derived slope variable (degrees) from Shuttle Radar Topography Mission (SRTM) elevation data which was accessed through the U.S. Geological Survey website. The spatial resolution of the data was approximately 24.9 m x 24.9 m grid scale. In ArcGIS, we calculated the average slope for each municipality at which spatial resolution the land use share variables are available for the present paper.

Soil dataset at 500 m x 500 m grid scale originates from the EU Joint Research Centre - European Soil Data Centre (ESDAC). The dataset includes soil texture and content of coarse fragment. Soil texture consists of percentage of clay, percentage of sand, and percentage of silt. Content of coarse fragment is defined as the percentage of materials >2.0 mm present in soils[9]. For our analysis, we include the average clay and sand proportions along with the average coarse fragment content (calculated in ArcGIS).

Crop prices are included in the analysis as a way to investigate the role of the market. Crop price data is available at country level but only for cereal, oilseeds and potato. Data was accessed from FAO Statistics.

Finally, we include a trend variable in the analysis as a proxy to other factors that might have influenced the change in the patterns of agricultural land uses over time (e.g. technological developments). We also include country dummy variables as a way to factor in differences between countries due to conditions that are specific to each country (e.g. agricultural and environmental policy differences).

Year	Denmark	Finland	Norway	Sweden	Total
1979	.	.	401	.	401
1981	.	.	.	275	275
1982	91	.	.	.	91
1983	91	.	.	.	91
1985	91	.	.	278	369
1987	89	.	.	.	89
1989	89	.	402	278	769
1990	.	.	.	278	278
1991	.	.	.	278	278
1992	.	.	.	279	279
1993	.	.	.	279	279
1994	.	.	.	280	280
1995	.	285	.	280	565
1997	.	285	.	.	285
1998	.	285	.	.	285
1999	87	.	404	283	774
2000	.	285	404	.	689
2001	.	285	404	.	689
2002	.	285	405	.	690
2003	.	285	404	284	973
2004	.	285	404	.	689
2005	.	285	404	285	974
2006	.	285	402	.	687
2007	.	285	401	285	971
2008	.	285	401	.	686
2009	.	285	402	.	687
2010	87	285	401	287	1060
2011	.	285	.	.	285
2012	.	285	.	.	285
Total	625	4560	5639	3929	14,753

Table S1. Number of observations: This table presents the annual total number of observations by country.

Variables	Definition
**Dependent variables**	
Cereal share	Land share of cereal planted fields
Grass share	Land share of grass planted fields
Oil seed share	Land share of oil seed rape planted fields
Others share	Land share of other agricultural land use
**Explanatory variables**	
Growing season length (GSL)	Annual number of days bounded by daily mean temperature of >5°C for 5 consecutive days and daily mean temperature <5°C for 5 consecutive days
GDD (degree days)	Sum of average daily temperature >5°C for all days in a in a given year's growing season GDD=Sum$(T_a - T_b)$ where T_a is average daily temperature and T_b is the baseline temperature
Precipitation(mm)	Total annual precipitation
Evapotranspiration(mm)	Total annual evapotranspiration
Soil moisture(mm)	Average soil moisture over the growing season
Slope(degrees)	Average slope of the agricultural area
Coarse Soil	Percentage of coarse soil in the field
Clay soil	Percentage of clay soil in the field
Sand soil	Percentage of sand soil in the field
Silt soil	Percentage of silt soil in the field
Cereal price(USD/tonne)	Price of cereal
Potato price(USD/tonne)	Price of potato
Oilseed price(USD/tonne)	Price of oil seed rape

Table S2. Variable definition. Notes: This table presents the description of our dependent and explanatory variables

	Mean	SD	Min.	Max.
Cereal share	0.33	0.26	0.00	1.00
Grass share	0.54	0.33	0.00	1.00
Oil seed share	0.02	0.04	0.00	0.72
Others share	0.11	0.12	0.00	1.00
GDD	932.48	363.96	6.64	1863.06
Precipitation	1036.68	438.82	556.75	3491.06
Evapotranspiration	475.58	141.44	67.21	1068.36
Soil moisture	368.94	97.02	60.36	636.10
Slope	5.91	5.61	0.10	31.90
Coarse Soil	13.94	3.71	6.09	25.50
Clay soil	11.13	5.47	2.31	30.15
Sand soil	61.02	9.73	34.88	87.09
Silt soil	27.85	6.38	8.01	47.45
Cereal price	213.10	80.46	94.74	421.54
Potato price	226.33	65.65	89.41	357.95
Oilseed price	459.66	179.09	162.39	813.21
Observations		14,753		

Table S3. Summary statistics. Notes: This table presents the summary statistics of the unbalanced panel data used in our multinomial fractional logit estimation.

Scenario	Variables	Mean	SD	Min.	Max.
Baseline(1981-2010)	GDD	963.36	390.24	15.69	1732.23
	Precipitation	1044.82	434.66	607.86	3246.20
	Evapotranspiration	482.50	141.33	76.71	984.22
	Soil moisture	365.63	96.67	61.50	634.76
RCP4.5(2041-2070)	GDD	1135.85	408.38	82.33	1971.44
	Precipitation	1128.51	434.73	642.41	3380.54
	Evapotranspiration	519.82	134.52	111.37	953.52
	Soil moisture	371.24	97.50	61.66	636.39
RC8.5(2041-2070)	GDD	1200.01	413.19	73.94	2038.69
	Precipitation	1163.60	417.10	677.98	3281.99
	Evapotranspiration	528.00	137.55	107.53	930.32
	Soil moisture	372.66	98.11	60.94	630.04
RCP4.5(2071-2100)	GDD	1220.88	420.49	98.90	2068.90
	Precipitation	1174.41	443.63	667.63	3500.42
	Evapotranspiration	532.63	134.67	113.73	945.24
	Soil moisture	370.85	97.60	62.49	642.38
RCP8.5(2071-2100)	GDD	1471.18	452.54	173.44	2415.63
	Precipitation	1230.85	460.87	739.28	3585.73
	Evapotranspiration	561.51	129.84	91.10	899.05
	Soil moisture	373.16	99.09	63.14	651.38
	Observations	1060			

Table S4. Summary statistics. Notes: This table presents the summary statistics of the baseline and projected climate data for the 1060 municipalities.

S.2 Results

In this section, we present estimation results, robustness and sensitivity tests as well as projection results.

Variables	Cereal	Grass	Oil seed
GDD	0.003268**	-0.002762**	0.005508***
	(2.47)	(-2.47)	(3.28)
GDD Square	-0.000001369**	1.496e-07	-0.000001413**
	(-2.52)	(0.36)	(-2.16)
Precipitation	0.006577***	0.001968***	0.005734***
	(8.46)	(3.04)	(2.71)
Precipitation Square	-0.000003084***	-4.764e-07**	-0.000002815**
	(-8.79)	(-2.28)	(-2.40)
Evapotranspiration	0.01308**	0.002953	0.007846
	(2.47)	(0.98)	(1.16)
Evapotranspiration Square	-0.00001618***	-5.708e-07	-0.00001018
	(-3.07)	(-0.23)	(-1.57)
Soil Moisture	0.01283***	-0.004703	0.007481**
	(3.60)	(-1.42)	(1.99)
Soil Moisture Square	-0.00001531***	0.000005527	-0.00001090**
	(-3.59)	(1.34)	(-2.39)
Slope	-0.1115***	0.03345	-0.1181***
	(-4.26)	(1.27)	(-3.15)
Coarse	-0.02656**	0.01463	-0.06474***
	(-2.08)	(0.80)	(-3.42)
Clay	0.02466**	-0.01862	0.03328**
	(2.33)	(-1.37)	(2.28)
Sand	0.001773	0.02997***	-0.01855**
	(0.28)	(3.96)	(-2.08)
Cereal Price	-0.001189**	-0.004061***	-0.004528***
	(-2.49)	(-7.51)	(-6.99)
Potato Price	0.001544***	0.001454***	0.0006737***
	(8.86)	(6.46)	(3.38)
Oilseed Price	-0.0003499	0.001664***	0.001975***
	(-1.59)	(6.73)	(6.51)
Constant	-60.369***	-120.09***	-11.562
	(-5.68)	(-9.95)	(-0.84)
Country dummies	Yes	Yes	Yes
Country linear trend	Yes	Yes	Yes
Observations	14753		
Log-PseudoLik.	-12037.1		

Table S5. Fractional Multinomial Logit Parameter Estimates. This table presents the estimation results of pooled multinomial fractional logit model. Fully robust clustered t-statistics are in parentheses. ***, ** and * denote significance on the 1%, 5% and 10% significance level, respectively.

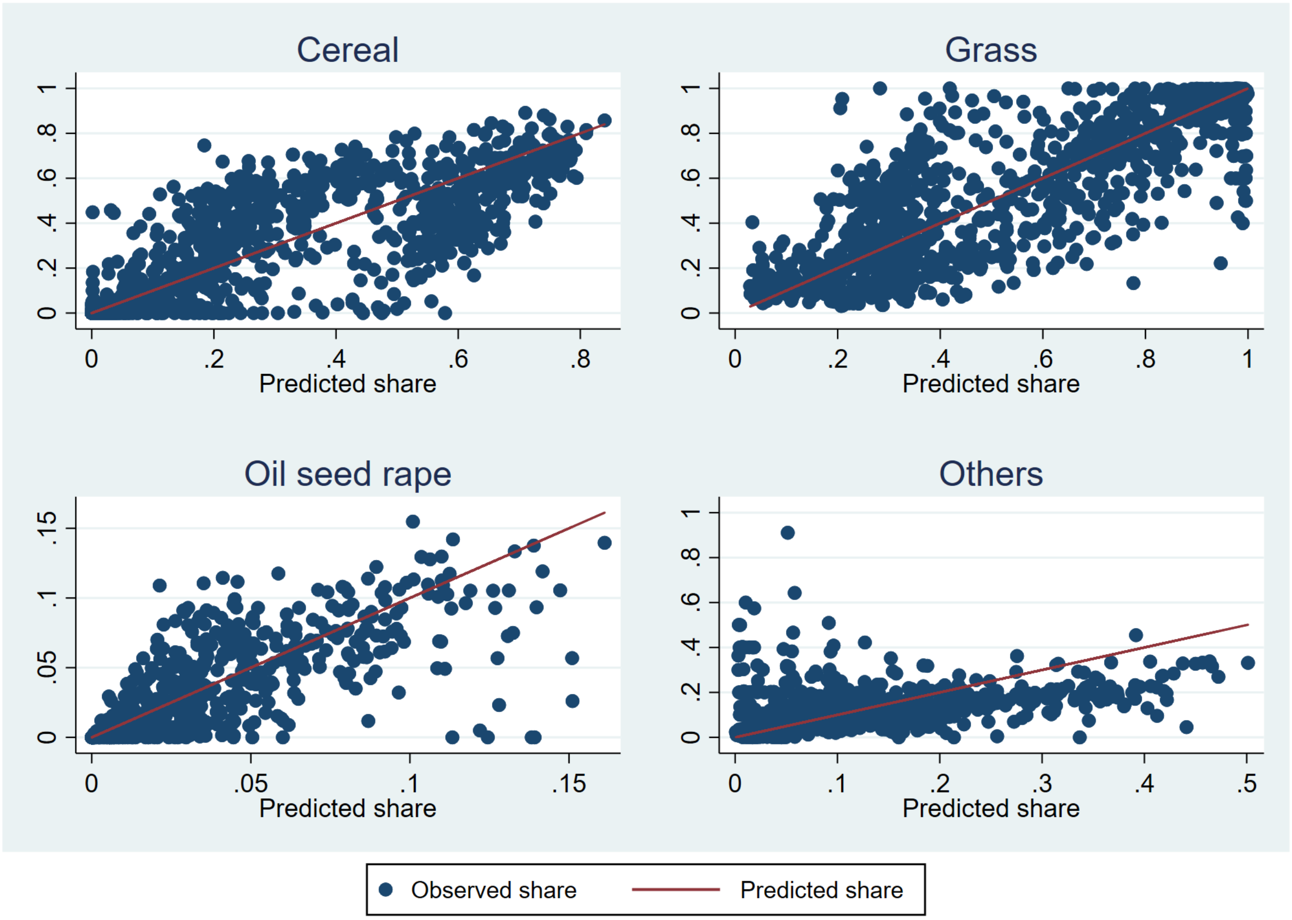

Figure S3. Model testing and robustness: We test the validity of the model using hold-out-sample in our data. We split the sample into two: estimation sample (1979 - 1999) and hold-out-sample (2000 - 2010). Then we calculate the time average of all our variables for the hold-out-sample and predict the average shares using the parameters from the estimation sample. The figures show scatter plots of the observed shares against the predicted shares for the hold-out-sample for 1062 municipalities. Moreover, we test whether the difference in means between the observed and predicted shares for the hold-out-sample are different from zero. The t-test results indicate that the mean difference for cereal, grass, and others are not statistically different even at 10%. For oil seed rape the difference is statistically different. However, this result is expected as oil seed rape is not produced in Norway and therefore has a share of zero in our data. Dropping observations with zero shares results in statistically insignificant difference in the shares. The results are sensitive to the choice of periods, but the magnitude of the difference between observed and predicted shares is small.

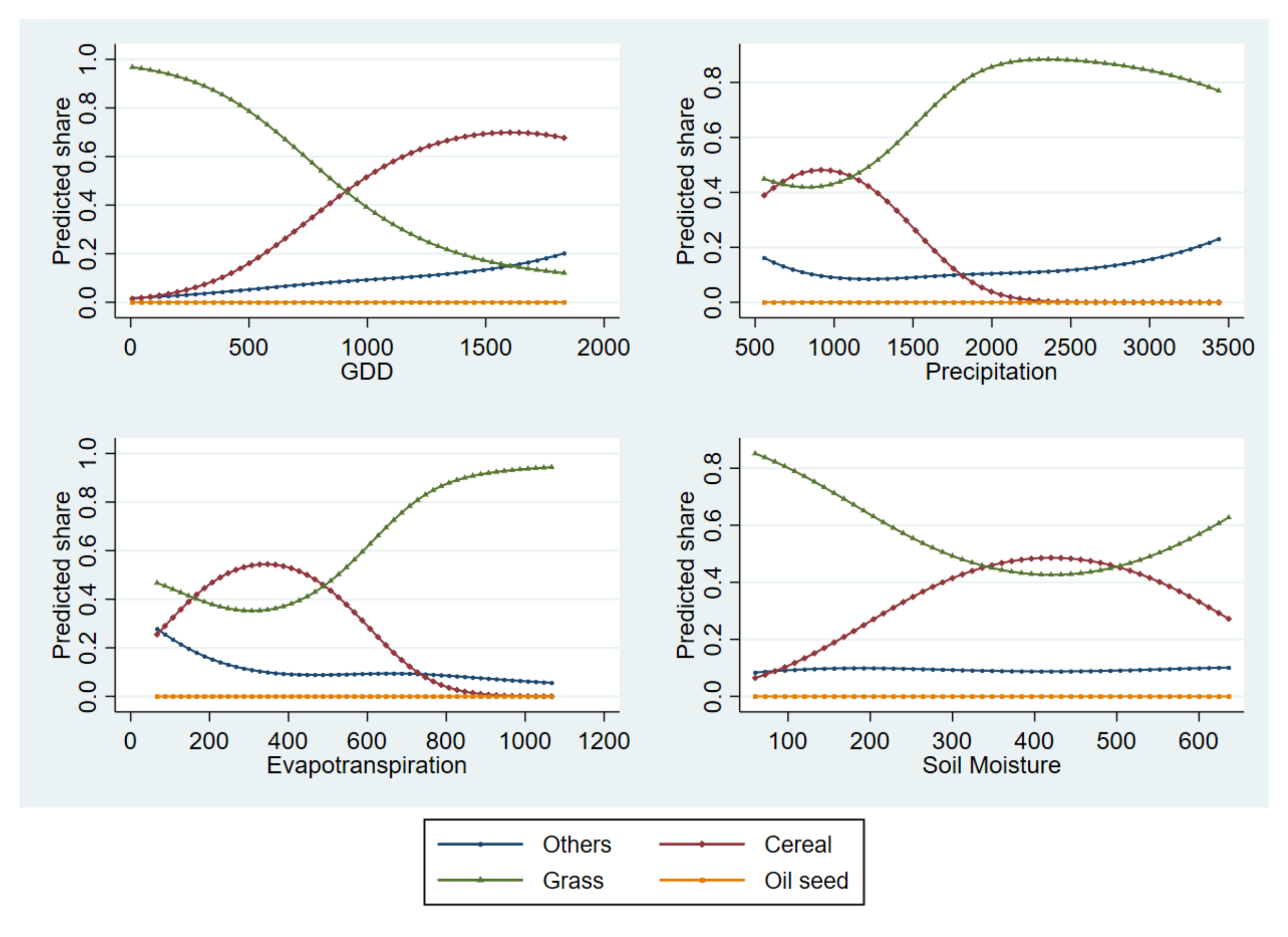

Figure S4. Predicted land use shares over the observed range of the climatic variables: The marginal effect at mean estimates provided in Table 1 in the main paper are calculated at the mean value of the variable in question while also keeping all the other variables in the model at their mean values. This figure presents the predicted shares of the different land use classes over the whole range of the climate variables (not just the mean) while keeping all other variables at their mean values. This means that we can also calculate the marginal effects over the entire range of the climatic variables (not just the mean). The marginal effect estimates can be calculated by differentiating the predicted shares with respect to the respective climatic variables over the entire range. Thus, at the rising section of the curves, the marginal effect is positive while it is negative at the declining section of the curve.

Sensitivity to climate projection uncertainties

Our future agricultural land use share prediction is carried out based on climate projections from a single climate model (RCA4). Thus, in order to account for the range of climate projections for the Nordic region, we attempted to re-estimate the predicted shares in Table 2 in the paper for the Nordic level by adding to or subtracting from their respective mean values a 5% and 10% of the standard deviation of the projected climate variables. These results reported in Table S6 are consistent with the results presented in Table 2 in the paper except for a small difference in magnitude. However, we recognize that the sensitivity analysis we have employed is somewhat arbitrary and potentially biased because it does not consider the range of projected climate from actual climate models and it is unlikely that the climate variables would move in the same direction or by the same proportion.

SD	Scenario	Cereal	Grass	Oilseed Rape	Others
-5%	RCP4.5(2041-2070)	0.0310***	-0.0526***	0.0000142	0.0216***
		(5.33)	(-7.19)	(0.40)	(6.08)
	RCP8.5(2041-2070)	0.0444***	-0.0751***	0.0000223	0.0307***
		(5.01)	(-6.75)	(0.40)	(5.56)
	RCP4.5(2071-2100)	0.0328***	-0.0711***	0.0000226	0.0382***
		(3.30)	(-5.74)	(0.39)	(6.24)
	RCP8.5(2071-2100)	0.0372	-0.144***	0.0000585	0.107***
		(1.46)	(-4.61)	(1.49)	(5.99)
+5%	RCP4.5(2041-2070)	0.0274***	-0.0540***	0.0000165	0.0266***
		(3.61)	(-5.69)	(0.37)	(5.79)
	RCP8.5(2041-2070)	0.0370***	-0.0741***	0.0000245	0.0370***
		(3.48)	(-5.54)	(0.38)	(5.56)
	RCP4.5(2071-2100)	0.0242**	-0.0695***	0.0000246	0.0452***
		(2.05)	(-4.69)	(0.37)	(6.07)
	RCP8.5(2071-2100)	0.0178	-0.138***	0.0000593	0.120***
		(0.65)	(-3.98)	(1.40)	(5.83)
-10%	RCP4.5(2041-2070)	0.0311***	-0.0506***	0.0000129	0.0196***
		(6.23)	(-7.97)	(0.42)	(6.25)
	RCP8.5(2041-2070)	0.0464***	-0.0744***	0.0000209	0.0279***
		(5.80)	(-7.38)	(0.41)	(5.56)
	RCP4.5(2071-2100)	0.0353***	-0.0706***	0.0000213	0.0353***
		(3.92)	(-6.27)	(0.40)	(6.32)
	RCP8.5(2071-2100)	0.0453*	-0.146***	0.0000576	0.101***
		(1.86)	(-4.94)	(1.54)	(6.05)
+10%	RCP4.5(2041-2070)	0.0239***	-0.0534***	0.0000175	0.0295***
		(2.81)	(-5.02)	(0.36)	(5.69)
	RCP8.5(2041-2070)	0.0316***	-0.0723***	0.0000254	0.0407***
		(2.75)	(-4.97)	(0.37)	(5.55)
	RCP4.5(2071-2100)	0.0183	-0.0675***	0.0000254	0.0492***
		(1.44)	(-4.20)	(0.35)	(5.99)
	RCP8.5(2071-2100)	0.00661	-0.135***	0.0000590	0.128***
		(0.23)	(-3.68)	(1.35)	(5.74)

Table S6. Predicted change in land use shares. This table presents a re-estimation of the predicted shares in Table 2 for the Nordic level by adding or subtracting a 5% and 10% of the standard deviation in the projected climate variables to their respective mean. z-statistics are given in parentheses. ***, ** and * denote significance on the 1%, 5% and 10% significance level, respectively.

	Baseline[a]	Differences from 1981-2010[b]			
	1981-2010	2041-2070		2071-2100	
Mean location/land use	Historical	RCP 4.5	RCP 8.5	RCP 4.5	RCP 8.5
		**Longitude**			
Cereal	4599874	31700	52236	47649	93050
Grass	4608791	-25613	-43765	-40314	-62418
Oil seed rape	4560816	13440	31987	32130	62525
Others	4614790	-37969	-53209	-50041	-81735
		**Latitude**			
Cereal	3947533	49504	82063	77812	152584
Grass	4122379	-30086	-60396	-51533	-68655
Oil seed rape	3832123	16993	40487	41846	83787
Others	3960528	-45986	-65177	-63236	-98870

Table S7. Baseline mean longitudes and latitudes and climate change induced predicted change in longitudes and latitudes. Notes: [a] Shows area weighted predicted coordinates based on the baseline observations. [b] Shows differences between historical averages and climate induced future averages.

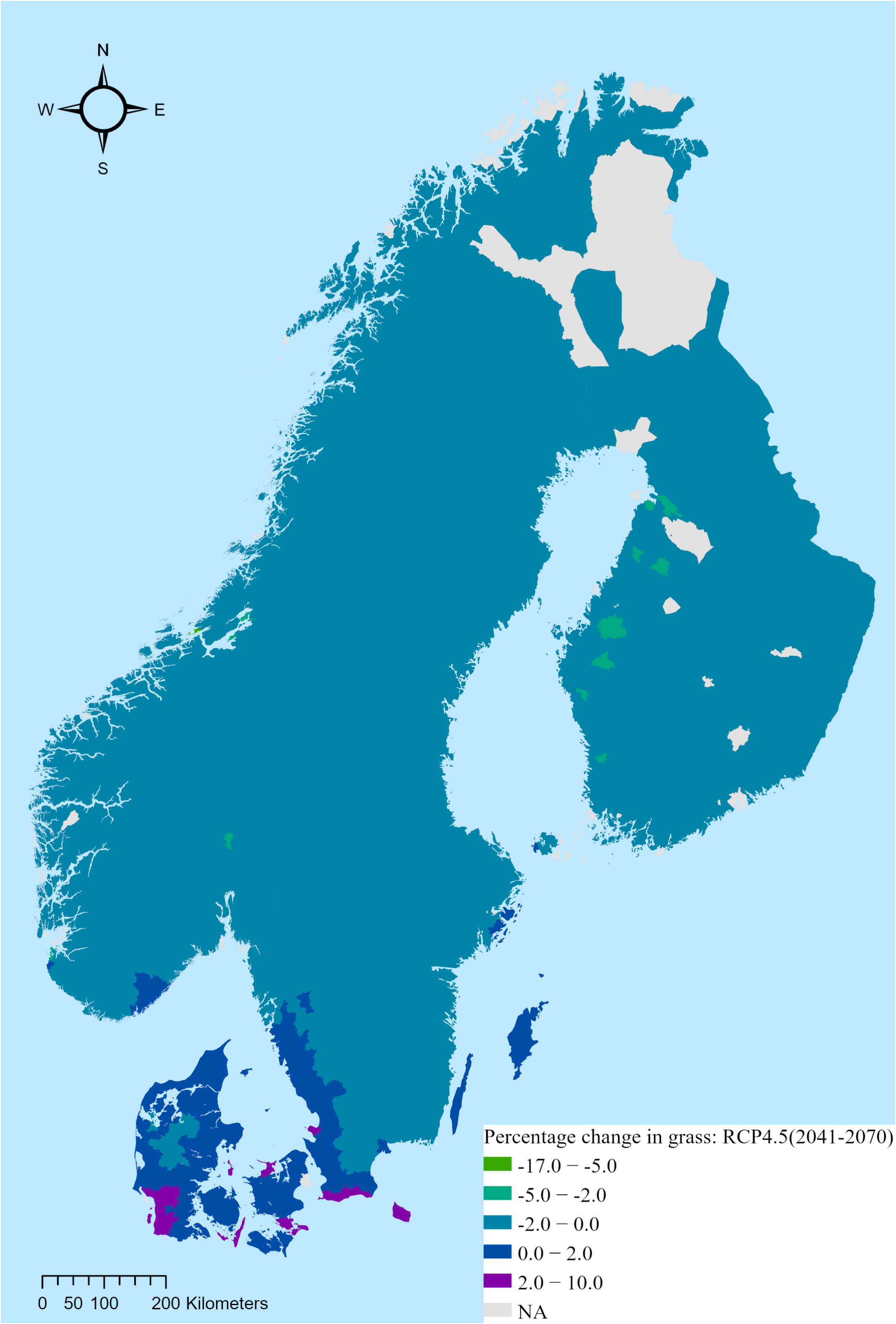

Figure S5. Predicted land use change for grass under the RCP4.5(2041-2070) climate scenario. The map presents a weighted municipality level percentage change in land use share of grass under the RCP4.5(2041-2070) climate relative to the baseline climate scenario. The map in this figure was generated in ArcGIS Pro 2.6.2 (https://www.esri.com/en-us/arcgis/products/arcgis-pro/overview)

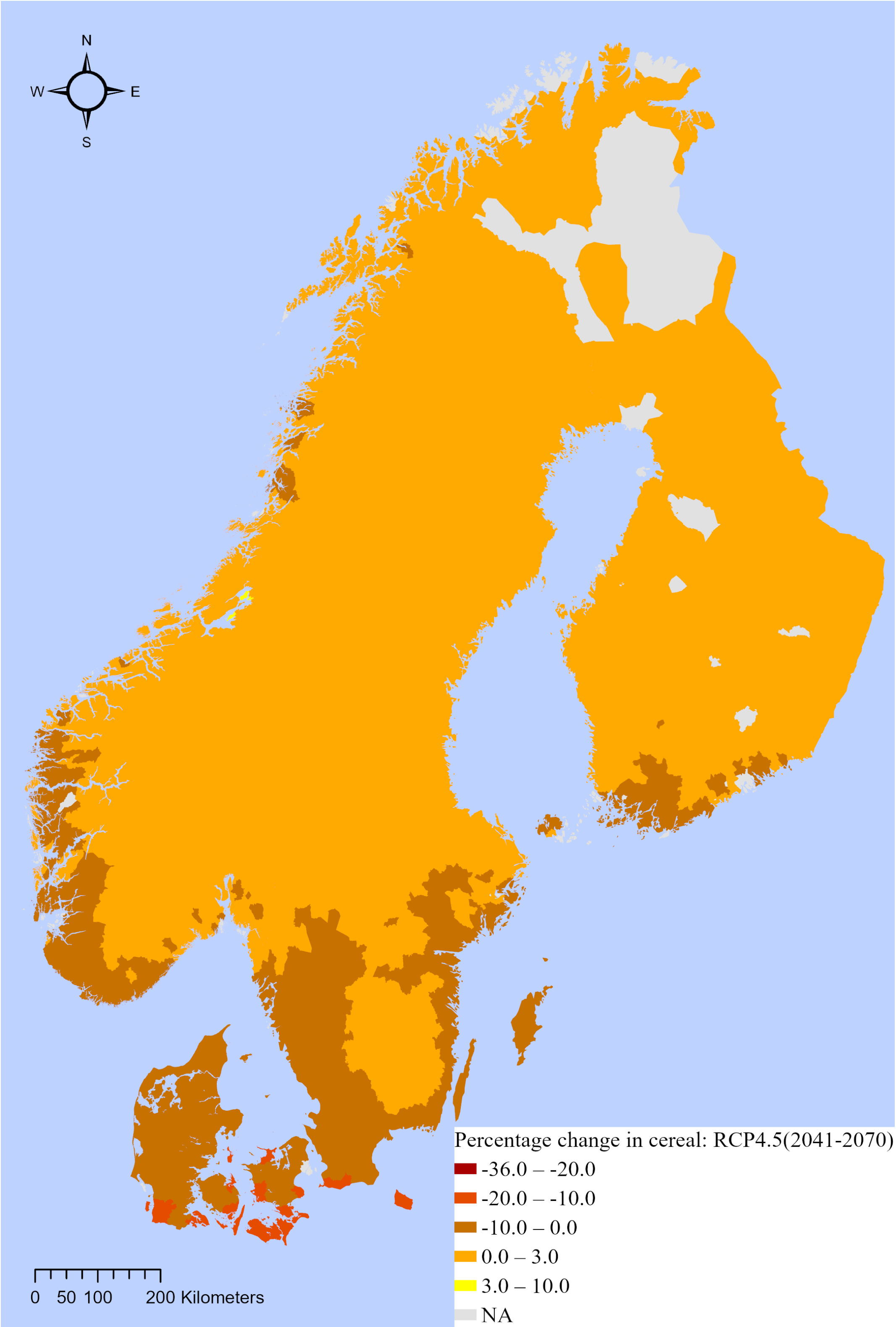

Figure S6. Predicted land use change for cereal under the RCP4.5(2041-2070) climate scenario. The map presents a weighted municipality level percentage change in land use share of cereal under the RCP4.5(2041-2070) climate relative to the baseline climate scenario. The map in this figure was generated in ArcGIS Pro 2.6.2 (https://www.esri.com/en-us/arcgis/products/arcgis-pro/overview)

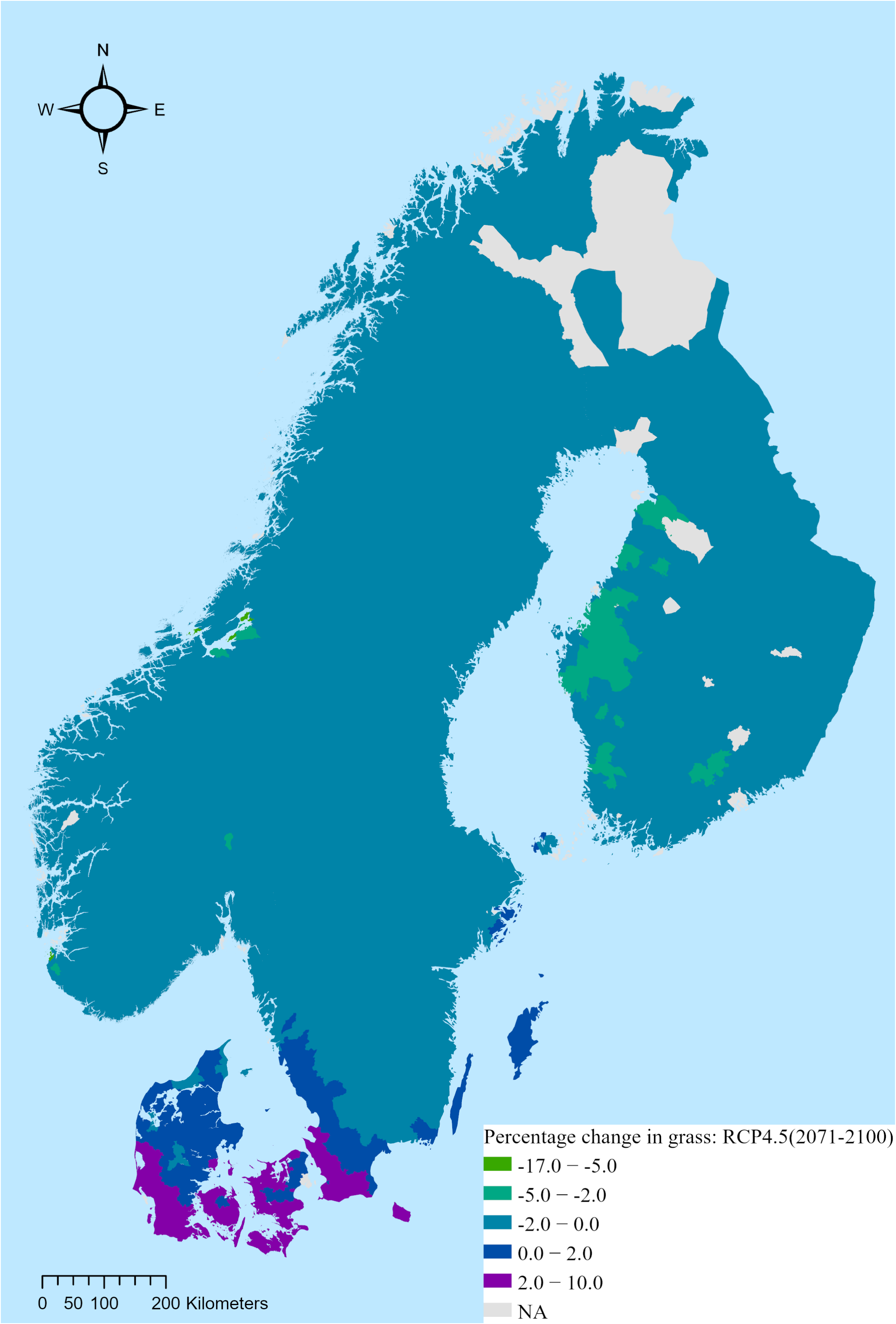

Figure S7. Predicted land use change for grass under the RCP4.5(2071-2100) climate scenario. The map presents a weighted municipality level percentage change in land use share of grass under the RCP4.5(2071-2100) climate relative to the baseline climate scenario. The map in this figure was generated in ArcGIS Pro 2.6.2 (https://www.esri.com/en-us/arcgis/products/arcgis-pro/overview)

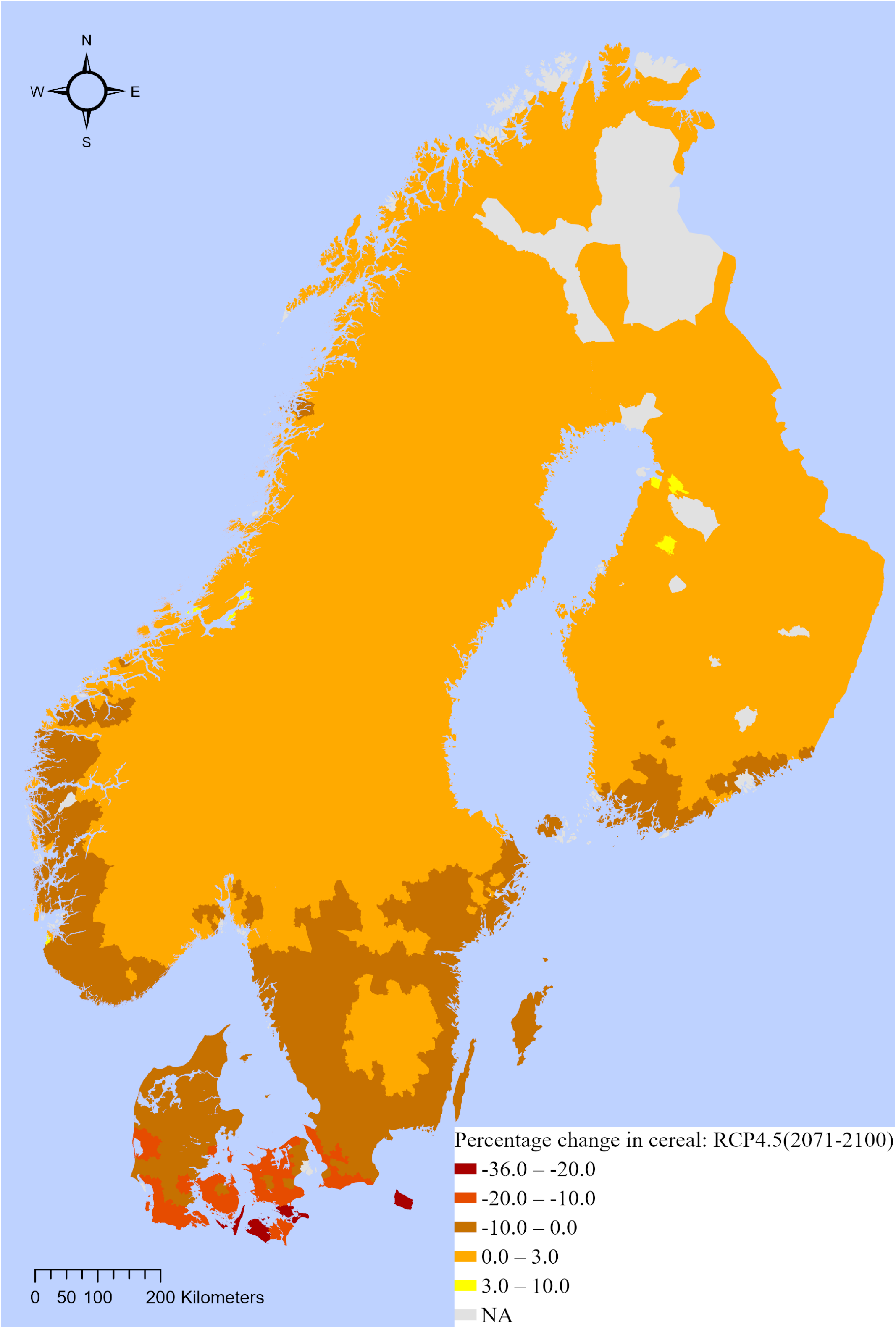

Figure S8. Predicted land use change for cereal under the RCP4.5(2071-2100) climate scenario. The map presents a weighted municipality level percentage change in land use share of cereal under the RCP4.5(2071-2100) climate relative to the baseline climate scenario. The map in this figure was generated in ArcGIS Pro 2.6.2 (https://www.esri.com/en-us/arcgis/products/arcgis-pro/overview)

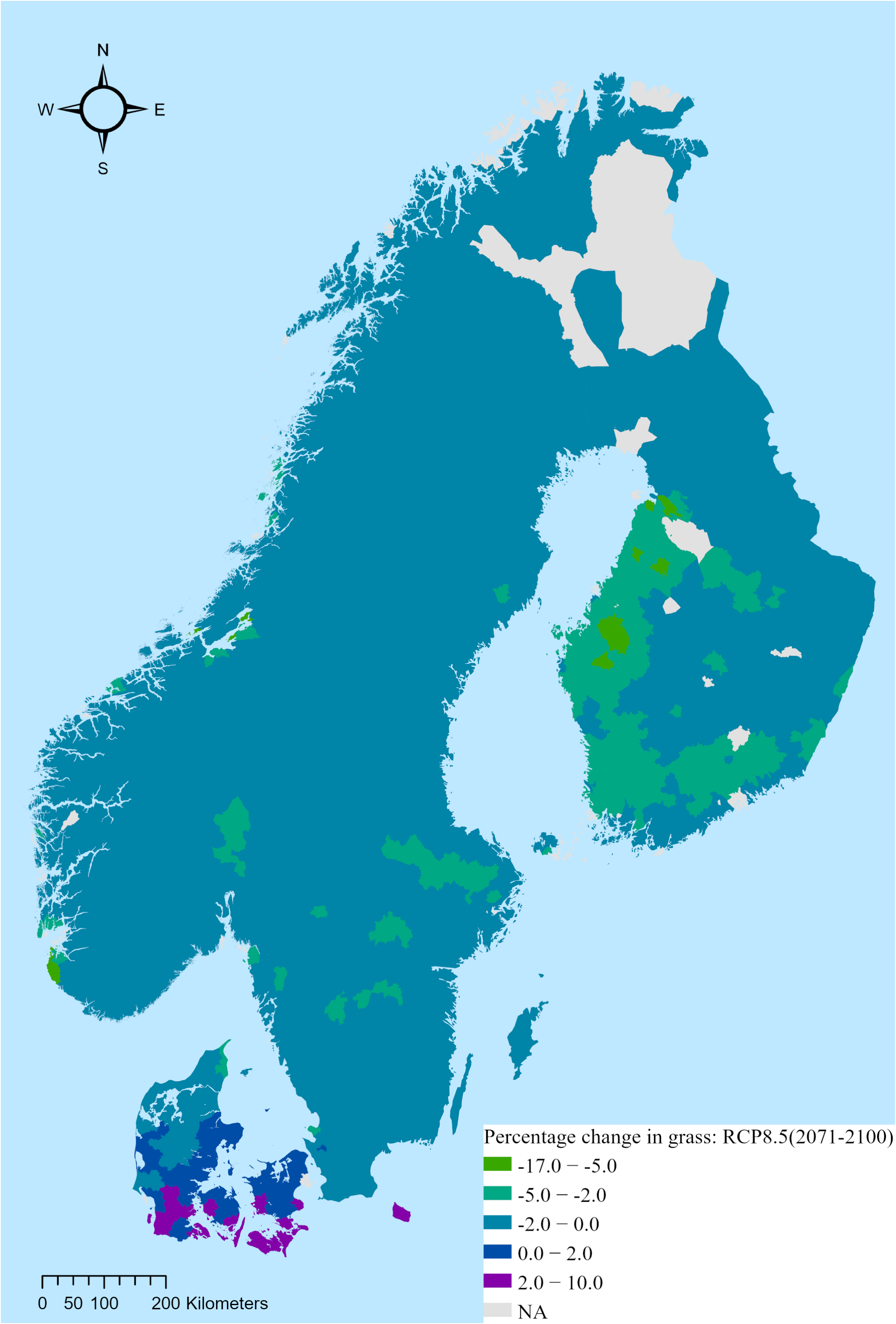

Figure S9. **Predicted land use change for grass under the RCP8.5(2071-2100) climate scenario.** The map presents a weighted municipality level percentage change in land use share of grass under the RCP8.5(2071-2100) climate relative to the baseline climate scenario. The map in this figure was generated in ArcGIS Pro 2.6.2 (https://www.esri.com/en-us/arcgis/products/arcgis-pro/overview)

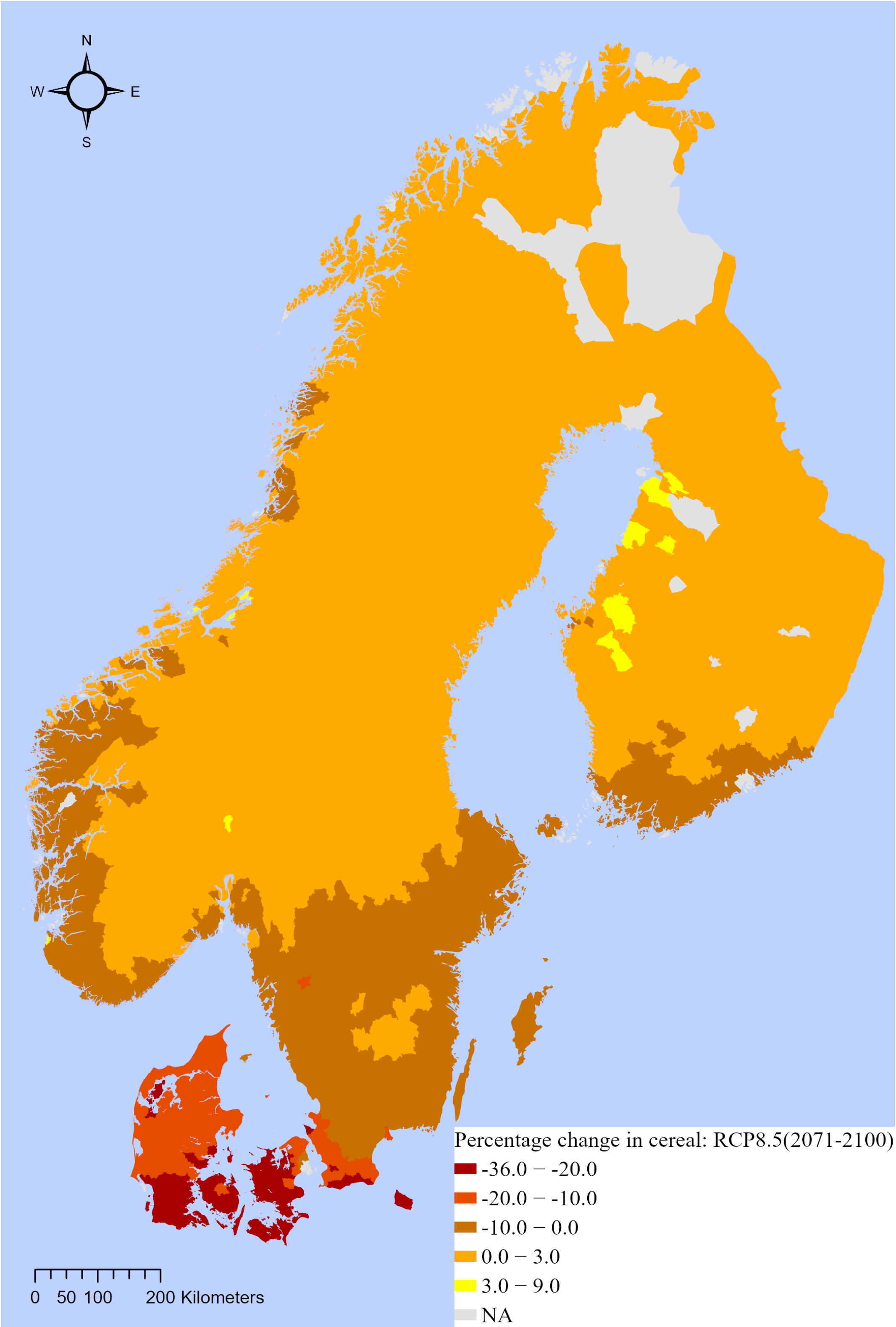

Figure S10. Predicted land use change for cereal under the RCP8.5(2071-2100) climate scenario. The map presents a weighted municipality level change in land use share of cereal under the RCP8.5(2071-2100) climate relative to the baseline climate scenario. The map in this figure was generated in ArcGIS Pro 2.6.2 (https://www.esri.com/en-us/arcgis/products/arcgis-pro/overview)

References

1. Mendelsohn, R., Nordhaus, W. D. & Shaw, D. The impact of global warming on agriculture: a Ricardian analysis. *The Am. economic review* 753–771 (1994).
2. Polsky, C. & Easterling III, W. E. Adaptation to climate variability and change in the us great plains:: A multi-scale analysis of ricardian climate sensitivities. *Agric. Ecosyst. & Environ.* **85**, 133–144 (2001).
3. Seo, S. N. & Mendelsohn, R. An analysis of crop choice: Adapting to climate change in south american farms. *Ecol. economics* **67**, 109–116 (2008).
4. Mu, J. E., Sleeter, B. M., Abatzoglou, J. T. & Antle, J. M. Climate impacts on agricultural land use in the usa: the role of socio-economic scenarios. *Clim. Chang.* **144**, 329–345 (2017).
5. Zhang, P., Zhang, J. & Chen, M. Economic impacts of climate change on agriculture: The importance of additional climatic variables other than temperature and precipitation. *J. Environ. Econ. Manag.* **83**, 8 – 31, DOI: https://doi.org/10.1016/j.jeem.2016.12.001 (2017).
6. Lubowski, R. N., Plantinga, A. J. & Stavins, R. N. Land-use change and carbon sinks: econometric estimation of the carbon sequestration supply function. *J. Environ. Econ. Manag.* **51**, 135–152 (2006).
7. Fezzi, C. & Bateman, I. J. Structural agricultural land use modeling for spatial agro-environmental policy analysis. *Am. J. Agric. Econ.* **93**, 1168–1188 (2011).
8. Lungarska, A. & Chakir, R. Climate-induced land use change in france: impacts of agricultural adaptation and climate change mitigation. *Ecol. Econ.* **147**, 134–154 (2018).
9. Ballabio, C., Panagos, P. & Monatanarella, L. Mapping topsoil physical properties at european scale using the lucas database. *Geoderma* **261**, 110–123 (2016).
